# Analysis of sequence diversity in *Plasmodium falciparum* glutamic acid-rich protein (PfGARP), an asexual blood stage vaccine candidate

**DOI:** 10.1038/s41598-023-30975-4

**Published:** 2023-03-09

**Authors:** Rattanaporn Rojrung, Napaporn Kuamsab, Chaturong Putaporntip, Somchai Jongwutiwes

**Affiliations:** 1 Molecular Biology of Malaria and Opportunistic Parasites Research Unit, Department of Parasitology, Faculty of Medicine, Chulalongkorn University, Bangkok, Thailand; 2 Medical Sciences Program, Faculty of Medicine, Chulalongkorn University, Bangkok, Thailand; 3 Community Public Health Program, Faculty of Health Science and Technology, Southern College of Technology, Nakorn Si Thammarat, Thailand

**Keywords:** Microbiology, Molecular biology

## Abstract

Glutamic acid-rich protein of *Plasmodium falciparum* (PfGARP) binds to erythrocyte band 3 and may enhance cytoadherence of infected erythrocytes. Naturally acquired anti-PfGARP antibodies could confer protection against high parasitemia and severe symptoms. While whole genome sequencing analysis has suggested high conservation in this locus, little is known about repeat polymorphism in this vaccine candidate antigen. Direct sequencing was performed from the PCR-amplified complete *PfGARP* gene of 80 clinical isolates from four malaria endemic provinces in Thailand and an isolate from a Guinean patient. Publicly available complete coding sequences of this locus were included for comparative analysis. Six complex repeat (RI-RVI) and two homopolymeric glutamic acid repeat (E1 and E2) domains were identified in PfGARP. The erythrocyte band 3-binding ligand in domain RIV and the epitope for mAB7899 antibody eliciting in vitro parasite killing property were perfectly conserved across isolates. Repeat lengths in domains RIII and E1-RVI-E2 seemed to be correlated with parasite density of the patients. Sequence variation in *PfGARP* exhibited genetic differentiation across most endemic areas of Thailand. Phylogenetic tree inferred from this locus has shown that most Thai isolates formed closely related lineages, suggesting local expansion/contractions of repeat-encoding regions. Positive selection was observed in non-repeat region preceding domain RII which corresponded to a helper T cell epitope predicted to be recognized by a common HLA class II among Thai population. Predicted linear B cell epitopes were identified in both repeat and non-repeat domains. Besides length variation in some repeat domains, sequence conservation in non-repeat regions and almost all predicted immunogenic epitopes have suggested that PfGARP-derived vaccine may largely elicit strain-transcending immunity.

## Introduction

Despite integrative control efforts, there were an estimated of 247 million malaria cases with 619,000 deaths in 2021 of which *Plasmodium falciparum* was the main causative agent^1,2^. The emergence and widespread of drug resistant parasites and insecticide resistant mosquitoes have impeded the progress toward sustainable reduction of morbidity and disease elimination in several endemic areas. As alternative strategy, vaccination could be an important means for adjunctive malaria control. To date, a remarkable progress has been envisaged for vaccines against pre-erythrocytic stages targeting the infective sporozoites and probably the liver stage parasites whereas vaccines against asexual blood stages including multiple merozoite antigens have been explored as potential vaccine candidates^3^.

The ~ 80 kDa glutamic acid-rich protein of *P. falciparum* (PfGARP) is highly expressed during trophozoite development^4^ and is detectable during schizogony of intraerythrocytic parasites^5^. The gene encoding PfGARP of the FC27 strain contains 2248 bp, characterized by a short 5′-exon encoding a signal peptide followed by a 214 bp intron and a second exon spanning 653 codons^6^. PfGARP is composed entirely of intrinsically disordered structure and repetitive low complexity sequences in which glutamic acid, lysine and aspartic acid constitute over half of all amino acid residues in the protein. Four complex repeat-containing regions, three of which were rich in lysine residues, and two homopolymeric glutamic acid repeats have been identified in this protein^6^. It has been shown that the lysine-rich repeats in PfGARP account for an indispensable module for targeting the protein to the periphery of the infected erythrocyte. Furthermore, in vitro mutagenesis has revealed that the length of the lysine-rich repeats in PfGARP is crucial for peripheral targeting efficiency^7^. Meanwhile, variation in length of lysine-rich repeat regions occurred in several laboratory strains of *P. falciparum* including 3D7, Dd2, HB3, IT and 7G8 strains^7^. Domain mapping of PfGARP has identified an immunogenic lysine-rich repeat region as a secreted ligand capable of binding to an ectodomain of erythrocyte band 3, an anion-exchanger in the red cell membrane, as a host receptor^5^.

PfGARP-derived synthetic peptides containing the erythrocyte-binding repeats conferred aggregation of erythrocytes akin to rosette formation, a phenomenon contributing to microvascular obstruction during the pathogenesis of complicated malaria^5^. Meanwhile, mouse anti-PfGARP antibody elicited significant inhibition of parasite growth in vitro*.* Consistently, anti-PfGARP antibodies purified from pooled plasma of Tanzanian adults could remarkably halt parasite growth in culture. *Aotus* monkeys immunized with PfGARP-derived vaccines were protected against high parasitemia and severe anemia^4^. Anti-PfGARP antibodies per se could mediate parasite killing by triggering programmed cell death in the asexual blood-stage parasites. Tanzanian children who mounted anti-PfGARP antibody responses upon natural infections had lower risk of severe malaria than those without detectable antibodies. Likewise, the levels of parasitemia in Kenyan adolescents and adults inversely associated with the magnitude of natural anti-PfGARP antibody responses^4^. Therefore, PfGARP is a promising target for anti-disease vaccine while it is also considered to be a potential marker for disease progression^3,4,8^.

Antigenic polymorphisms in malarial vaccine candidates could hinder an effective vaccine design if the protective immunity is predominantly strain-specific^9^. Although it has been suggested that PfGARP exhibited meager genetic diversity, the conclusion seemed to be mainly drawn from whole genome sequence data where variation in the repetitive sequences requires further elucidation^4,10^. Herein, we analyzed the nucleotide sequences of this locus among *P. falciparum* populations from four major malaria endemic areas of Thailand. Results revealed limited sequence variation in non-repeat regions of PfGARP among Thai and global isolates whereas differential diversity in repeat domains was observed. In addition to previously identified four repeat-encoding domains and two homopolymeric glutatamic acid repeat regions^6^, two additional regions have been newly recognized to possess repetitive sequences. Furthermore, parasite genetic structure and in silico prediction of immunogenic epitopes in PfGARP have been analyzed.

## Results

### Genetic diversity and structural organization of PfGARP

**Figure 1:**
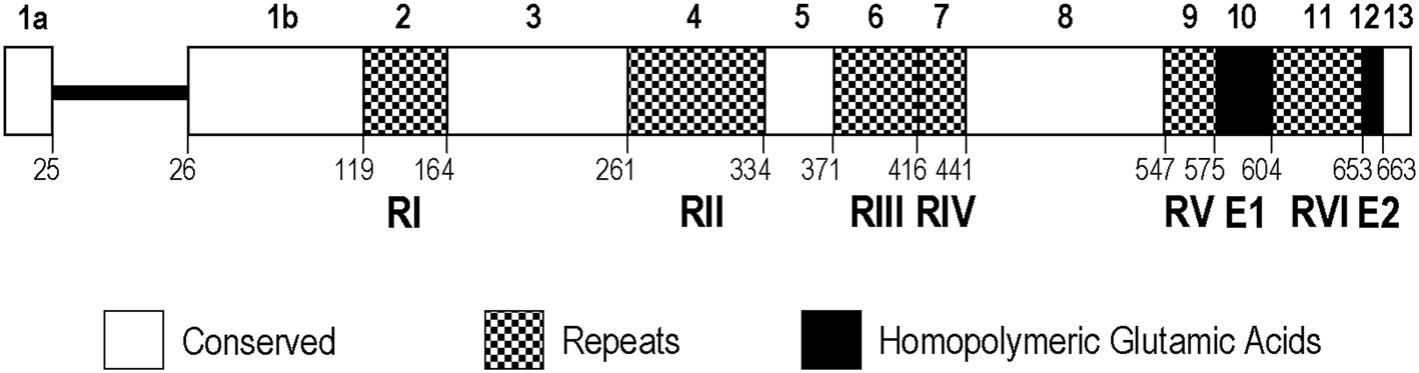
Schematic representation of *PfGARP*. Exons are shown as boxes and an intron as a dense line. Boxes are numbered above the scheme. Exons are characterized by conserved, repeats and homolymeric glutamic acids regions. Amino acid position at the end of each block/domain is indicated beneath the scheme. The nucleotide positions for boxes Ia, Ib and II to XIII are 1–75, 290–571, 572–706, 707–997, 998–1215, 1216–1327, 1328–1462, 1463–1537, 1538–1855, 1856–1939, 1940–2026, 2027–2173, respectively (positions corresponding to coding sequence of the FC27 strain, GenBank accession no. J03998).

The *PfGARP* sequences were successfully obtained from all 80 isolates which revealed clear and non-superposed signals on electropherograms. Size variation in *PfGARP* was observed among Thai isolates, ranging from 2179 to 2284 bp. In total, 26 alleles of *PfGARP* were identified among Thai isolates whereas an isolate (MDCU32) from a Guinean patient analyzed in this study had a different sequence. Likewise, size and sequence variation were also observed among previously reported sequences of this locus among isolates from other malaria endemic areas (n = 18) including African, Indochina, South American and Western Pacific countries, all of which possessed distinct sequences with size variation from 2209 to 2266 bp (Supplemental Table S1). Together with previously reported complete coding sequences, 44 haplotypes were identified (Table 1). Of these, 26 haplotypes were found among 80 Thai isolates (Supplemental Fig. S1) in which the numbers of haplotypes and haplotype diversity of isolates from Tak, Ubon Ratchathani and Chanthaburi Provinces were remarkably higher than those of Yala Province (Table 1). Likewise, nucleotide diversity of *P. falciparum* population from Yala Province was significantly lower than those of other endemic areas (Table 1). Based on available 99 complete coding sequences, *PfGARP* can be divided into 13 blocks consisting of five non-repeat and eight repeat-containing regions (Fig. 1).

**Table 1:** Haplotype and nucleotide diversity of *PfGARP* among Thai and global isolates.

Population	n	M	H	*h* ± SD	π ± SE
Thai	80	45	26	0.887 ± 0.022	0.00366 ± 0.00100
Tak	20	22	9	0.795 ± 0.087	0.00349 ± 0.00091
Ubon Ratchathani	20	32	8	0.868 ± 0.049	0.00365 ± 0.00082
Chanthaburi	20	35	11	0.868 ± 0.057	0.00333 ± 0.00081
Yala	20	1	2	0.100 ± 0.088	0.00005 ± 0.00005#
Non-Thai	19	52	19	1.000 ± 0.017	0.00576 ± 0.00083
Global	99*	58	44	0.925 ± 0.016	0.00424 ± 0.00098

*Complete gene sequences.

*M* the number of mutation sites, *H* the number of haplotypes, *h* haplotype diversity, *π* nucleotide diversity, *SD* standard deviation, *SE* standard error.

Test of the hypothesis that π for one province equals π for another province in Thailand: # *p* < 0.0005.

### Exon I and intron

The 75-bp coding region in exon I exhibited perfect sequence identity among Thai and worldwide isolates (block 1a in Fig. 1). The adjacent intron region displayed two variants due to short insertion/deletion of TA residues: one possessed 214 bp and the other contained 216 bp. The former was more prevalent among Thai and worldwide isolates accounting for 80% and 83.3%, respectively.

### Non-repeat regions in exon II

The nonrepetitive sequences in exon II were highly conserved containing two synonymous substitutions: c. 439A>G (E75) and c. 502A>T (I96) in block 1b and a synonymous substitution: c. 2248C>T (I678) in block 13 (positions corresponding to coding sequence of the FC27 strain, GenBank accession no. J03998) (Fig. 1). Five nonsynonymous substitutions occurred in conserved block 3 among non-Thai isolates: c. 707A>G (K165E), c.781G>T (D193Y), c. 852C>T (P213L), 854T>G (Y214D) and c. 861A>G (Y216C). The distribution of these single nucleotide polymorphisms (SNPs) among isolates is shown in Supplemental Table S2. The remaining non-repeat regions including blocks 5 and 8 were perfectly conserved among Thai and worldwide isolates.

### Repeat domain I (block 2)

Besides previously known four repeat blocks, two additional repeat regions have been identified in *PfGARP* based on Tandem Repeat Finder Program. Herein, these domains were assigned as repeat domains I-VI (RI-RVI) in which previously reported repeat sequence motifs in blocks 1–4 are corresponding to domains RI, RIII, RIV and RVI, respectively^6^. Analysis of 80 Thai and 18 worldwide isolates have shown that domain RI could be assigned to 13 alleles, characterized by KKX motif where X is D, K, E or H as previously described^6^. The tripeptide repeats in this domain varied from 12 to 19 units. Designation of alleles in all repeat-containing regions was referred here according to the number of amino acid residues. When different sequences contained identical number of amino acids, alleles were further subdivided by adding an alphabet following the number to indicate variants; thereby, new alleles could be included in alphabetical order. Of these, eight alleles occurred in Thai isolates which included RI-57, RI-51A, RI-51B, RI-51C, RI-48, RI-45A and RI-45B. The RI-45A allele was most prevalent and seemed to circulate across endemic provinces in Thailand (Table 2).

**Table 2:** Diversity and distribution of repeat alleles in block II (repeat domain I) of PfGARP.

Allele	Amino acid sequence	Thai isolates, n					Non-Thai isolates/strains^#^	Total
		Tak	Ubon Ratchathani	Chanthaburi	Yala	Total		
RI-57	KKDKKEKKHKKDKKEKKEKKDKKEKKDKKEKKDKKEKKDKKEKKDKKEKKDKKDKKK			1		1		
RI-51A	KKDKKEKKHKKDKKEKKEKKDKKEKKDKKEKKDKKEKKDKKEKKDKKDKKK	1	8	2		11	KH2	1
RI-51B	KKDKKEKKHKKDKKEKKEKKDKKEKKDKKEKKDKKEKKHKKEKKHKKDKKK	1		1		2	KH1	1
RI-51C	KKDKKEKKHKKDKKDKKEKKDKKEKKDKKEKKDKKEKKDKKEKKHKKDKKK	1				1		
RI-51D	KKDKKEKKHKKDKKEKKEKKDKKEKKDKKEKKDKKEKKDKKEKKHKKDKKK						IGH-CR14	1
RI-51E	KKDKKEKKHKKDKKEKKEKKDKKEKKDKKEKKHKKEKKHKKEKKHKKDKKK						MDCU32, GA01, GB4, SN01, TG01	5
RI-51F	KKDKKEKKHKKDKKEKKEKKEKKEKKDKKEKKDKKEKKHKKEKKHKKDKKK						FCC1/HN	1
RI-51G	KKDKKEKKHKKDKKEKKEKKDKKEKKDKKEKKHKKEKKHKKDKKHKKDKKK						ML01	1
RI-48	KKDKKEKKHKKDKKEKKEKKEKKDKKEKKDKKEKKHKKEKKHKKDKKK		1			1		
RI-45A	KKDKKEKKHKKDKKEKKEKKDKKEKKDKKEKKHKKEKKHKKDKKK	15	11	15	20	61	3D7, FC27, IT, KE01, CD01, UGT5.1	6
RI-45B	KKDKKEKKHKKDKKDKKEKKDKKEKKDKKEKKDKKEKKDKKDKKK	2		1		3	Dd2	1
RI-45C	KKDKKEKKHKKDKKEKKEKKDKKEKKHKKEKKHKKEKKHKKDKKK						HB3	1
RI-36	KKDKKEKKHKKDKKEKKDKKEKKHKKEKKHKKDKKK						SD01	1
	Total	20	20	20	20	80		19

MDCU32 is from a Guinean patient.

^#^GenBank accession numbers are listed in “Materials and methods”.

### Repeat domain II (block 4)

Repeat domain II has been newly identified in this study, characterized by two copies of degenerate 33-codons encoding KKERKQKEKEMKE(or K)QE(or K) KIEKK(or E)K(or R)KKQ(or K)EEKEKKKQ(or K)E (or K) intervened by a short region encoding KERKKQE. The sequence of repeat domain II exhibited sequence conservation except a deletion of the last two lysine residues in all Thai and most worldwide isolates (Supplemental Table S3).

### Repeat domain III (block 6)

Repeat domain III, characterized by degenerate pentapeptide motifs encoding E(or G/K)EH(or D)K(or E/K)E(or K/S) in which the repeats comprised EEHKE, GEHKE, GEDKE, GEHKK, EEHKK, GEHEE, EEHKS, GEHKS and KEHKE. In total 19 alleles were identified, 11 of which were found in Thailand. Allele RIII-30 was most common and could be detected in all isolates from Yala Province, followed by alleles RIII-45A and RIII-35A whereas the isolate MDCU32 had a unique sequence (Table 3).

**Table 3:** Diversity and distribution of repeat alleles in block 6 (repeat domain III) of PfGARP.

Allele	Amino acid sequence	Thai isolates, n					Non-Thai isolates/strains*	Total
		Tak	Ubon Ratchathani	Chanthaburi	Yala	Total		
RIII-45A	EEHKEGEHKEEEHKEGEDKEGEDKEGEHKKEEHKKEEHKSKEHKS	3	10	4		17	KH1, KH2	2
RIII-45B	EEHKEGEHKEEEHKEEEHKEEEHKKEEHKKEEHKKEEHKSKEHKS	3	3			6		
RIII-45C	EEHKEGEHKEEEHKEGEHKEGEHKEGEHKEGEHKEEEHKSKEHKS	3				3	IT	1
RIII-45D	EEHKEGEHKEEEHKEGEHKEGEHKEGEHKKEEHKKEEHKSKEHKS		2	1		3	FCC1/HN	1
RIII-45E	EEHKEGEHKEEEHKEEEHKEGEHKEGEHKKEEHKKEEHKSKEHKS			1		1		
RIII-45F	EEHKEGEHKEEEHKEGEHKEGEHKEEEHKEEEHKKEEHKSKEHKS						3D7, FC27, SD01	3
RIII-45G	EEHKEGEHKEGEHKEEEHKEGEHKEGEHKEGEHKEEEHKSKEHKS						KE01	1
RIII-40A	EEHKEGEHKEEEHKEGEHKEGEHKEGEHKKEEHKSKEHKS			6		6		
RIII-40B	EEHKEGEHKEEEHKEEEHKEEEHKKEEHKKEEHKSKEHKS	2		1		3	Dd2	1
RIII-40C	EEHKEGEHEEGEHKEEEHKEGEHKEGEHKEEEHKSKEHKS						GA01, GB4, HB3, SN01	4
RIII-40D	EEHKEGEHKEEEHKEGEHKEEEHKEEEHKKEEHKSKEHKS						CD01, TG01	2
RIII-40E	EEHKEGEHEEGEHKEEEHKEGEHKSKEHKEEEHKSKEHKS						IGH-CR14	1
RIII-40F	EEHKEGEHKEGEHKEEEHKEGEHKEGEHKEEEHKSKEHKS						ML01	1
RIII-40G	EEHKEGEHKEEEHKEGEHKEGEHKEEEHKEGEHKSKEHKS						MDCU32	1
RIII-35A	EEHKEGEHKEEEHKEGEHKEGEHKEEEHKSKEHKS	9	2	6		17		
RIII-35B	EEHKEGEHKEEEHKEEEHKEEEHKKEEHKSKEHKS		3			3		
RIII-35C	EEHKEGEHKEEEHKEGEHKEEEHKEGEHKSKEHKS			1		1		
RIII-35D	EEHKEGEHKEEEHKEGEHKEEEHKEEEHKKEEHKS						UGT5.1	1
RIII-30	EEHKEGEHKEEEHKEGEHKSKEHKSKEHKS				20	20		
	Total	20	20	20	20	80		19

MDCU32 is from a Guinean patient.

*GenBank accession numbers are listed in “Materials and methods”.

### Repeat domain IV (block 7)

It has been shown that repeat domain IV (RIV) of PfGARP is a parasite ligand for human erythrocyte band 3 that could contribute to the cytoadherence during asexual blood stage development of *P. falciparum*^5^. Although RIV was located adjacent to RIII, the sequences were different in which the latter comprised 5 copies of degenerate pentapeptide repeats KGKKX where X was D, K, E or H as previously noted^6^. Analysis of Thai and worldwide isolates has shown perfect sequence identity in this domain, resulting in a single haplotype of this domain.

### Repeat domain V (block 9)

The newly recognized repeat domain V (RV) was characterized by imperfect repeats encoding KEVE(or Q)EE(or gap)S(or gap), flanked by EEDKKEES and DEEEVEED at the N- and C-termini of this domain, respectively. Five alleles have been identified in which the C-terminal sequence of allele RV-23 had a deletion of five codons encoding EEVEE. Of these, four alleles have been detected among Thai isolates (Table 4).

**Table 4:** Diversity and distribution of repeat alleles in block 9 (repeat domain V) of PfGARP.

Allele	Amino acid sequence	Thai isolates, n					Non-Thai isolates/strains*	Total
		Tak	Ubon Ratchathani	Chanthaburi	Yala	Total		
RV-35	EEDKKEESKEVEEESKEVQEESKEVQEDEEEVEED						FC27, ML01	2
RV-28A	EEDKKEESKEVEEESKEVQEDEEEVEED	10	18	5		33	3D7, FCC1/HN, GB4, HB3, IGH-CR14, IT, KE01,KH1, KH2, MDCU32, TG01, UGT5.1	12
RV-28B	EEDKKEESKEVQEESKEVQEDEEEVEED			2		2	Dd2, GA01, SD01, SN01	4
RV-23	EEDKKEESKEVEEESKEVQEDED			7		7	CD01	1
RV-21	EEDKKEESKEVQEDEEEVEED	10	2	6	20	38		
	Total	20	20	20	20	80		19

MDCU32 is from a Guinean patient.

*GenBank accession numbers are listed in “Materials and methods”.

### Repeat domain VI (block 11)

Repeat domain VI (RVI) contained degenerate heptapeptide repeats consisting of E(or D)E(or D)E (or D)XE(or D)E(or D)E(or D) where X is A, V, D, E or gap, followed by (E)_n_(D)_m_ residues where n and m varied from 1–5 to 1–3, respectively^6^. This repeat domain was the most polymorphic region in PfGARP, containing 27 alleles; 13 of these occurred among Thai isolates (Table 5). The isolate MDCU32 shared the same allele of this domain with the strain TG01 sequence from Togo (GenBank accession no. LR131450).

**Table 5:** Diversity and distribution of repeat alleles in block 11 (repeat domain VI) of PfGARP.

Allele	Amino acid sequence	Thai isolates, n					Non-Thai isolates/strains*	Total
		Tak	Ubon Ratchathani	Chanthaburi	Yala	Total		
RVI-74	EDEVEEDEDDAEEDEDDAEEDEDDAEEDEDDAEEDEDDAEEDEDDAEEDDDDAEEDDDEEDDDEDEEDEEEEED	2				2		
RVI-69	EDEVEEDEDDAEEDEDDAEEDEDDAEEDEDDAEEDEDDAEEDDDDAEEDDDDAEEDDDEDEEDEEEEED		5			5		
RVI-68	EDEVEEDEDDAEEDEDDAEEDEDDAEEDEDDAEEDEDDAEEDDDDAEEDDDEEDDDEDEDEDEEDEED						GB4	1
RVI-67	EDEVEEDEDDAEEDEDDAEEDEDDAEEDEDDAEEDEDDAEEDDDDAEEDDDEEDDDEDEEDEEEEED	2	9	3		14	IT, KH2	2
RVI-66	EDEVEEDEDDAEEDEDDAEEDEDDAEEDEDDAEEDEDDAEEDDDDAEEDDDEEDDDEDEDEEDEED						HB3	1
RVI-65A	EDEVEEDEDDAEEDEDDAEEDEDDAEEDEDDAEEDEDDAEEDDDEEDDDEEDDDEDEEDEEEEED	2				2		
RVI-65B	EDEVEEDEDDAEEDEDDAEEDEDDAGEDEDDAEEDEDDAEEDDDDAEEDDDEEEDVEDEEDEEED						FCC1/HN	1
RVI-63A	VEEDEDDAEEDEDEDDAEEDEDEDDAEEDEDDAEEDEDDAEEDDDDAEEDDDDAEEDDDEDED						KE01	1
RVI-63B	EDEVEEDEDDAEEDEDDAEEDEDDAEEDEDDAEEDDDDAEEDDDDAEEDDDEDEDEDEEDEED						TG01, MDCU32	2
RVI-61A	EDEVEEDEDDAEEDEDDAEEDEDDAEEDEDDAEEDDDDAEEDDDEEDDDEDEDEDEEDEED			7		7	UGT5.1	1
RVI-61B	EDEVEEDEDDAEEDEDDAEEDEDDAEEDEDDAEEDDDDAEEDDDDAEEDDDEDEDEEEEED						SD01	1
RVI-59A	EDEVEEDEDDAEEDEDDAEEDEDDAEEDEDDAEEDDDDAEEDDDEEEDEEEDDEDEDED			1		1		
RVI-59B	EDEDDAEEDEDDAEEDEDDAEEDEDDAEEDEDDAEEDDDDAEEDDDEDEEEDDEDEDED		2			2		
RVI-58A	EDEVEEDEDDAEEDEDDAEEDEDDAEEDEDDAEEDDDEEDDDEEDDDEDEEDEEEEED			1		1		
RVI-58B	VEEDEDDAEEDEDDAEEDEDDAEEDEDDAEEDDDDAEEDDDEEDDDEDEDEDEEDEED						GA01	1
RVI-58C	EDEVEEDEDDAEEDEDDAEEDEDDAEEDEDDDEEDDDEEDDDEEDDDEDEEDEEEEED		2			2		
RVI-57	EDEVEEDEDDAEEDEDDAEEDEDDAEEDEDDAEEDDDDAEDDDDAEEDDDEDEDEED						ML01	1
RVI-56A	EDEDDAEEDEDDAEEDEDDAEEDEDDAEEDDDDAEEDDDEEDDDEDEDEDEEDEED	4				4		
RVI-56B	EDEVEEDEDDAEEDEDDAEEDDDDAEEDDDDAEEDDDDAEEDDDDDEDEEEDDEED						KH1	1
RVI-56C	EDEVEEDEDDAEEDEDDAEEDEDDAEEDDDDAEEDDDDAEEDDDEDEDEDEEEEED						3D7	1
RVI-56D	VEEDEDDAEEDEDDAEEDEDDAEEDEDDAEEDDDDAEEDDDEEDDDEDEDEDEDED						IGH-CR14	1
RVI-55	EDEVEEDEDDAEEDEDDAEEDEDDAEEDEDDAEEDDDDAEEDDDDAEEDDDEDED			1		1	Dd2	1
RVI-54A	EDEVEEDEDDAEEDEDDAEEDEDDAEEDDDDAEEDDDEEDDDEDEDEDEEDEED			1		1		
RVI-54B	VEEDEDDAEEDEDDAEEDEDDAEEDEDDAEEDDDEEDDDEEDDDEDEDEEDEED						SN01	1
RVI-53	EDEVEEDEDDAEEDEDDAEEDEDDAEEDDDDAEEDDDDAEEDDDEDEDEDEED						CD01	1
RVI-51	EDEVEEDEDDAEEDEDDAEEDEDDAEEDDDDAEEDDDEEDDDEDEDEDEED	10	2	6	20	38		
RVI-49	EDEDEEDEDDAEEDEDDAEEDEDDAEEDDDEEDDDEEDDDEDEDEDEED						FC27	1
	Total	20	20	20	20	80		19

MDCU32 is from a Guinean patient.

*GenBank accession numbers are listed in “Materials and methods”.

### Homopolymeric glutamic acid repeats

Size variation was observed in homopolymeric glutamic acid repeats of domains E1 and E2, corresponding to blocks 10 and 12, respectively. The E1 domain contained 16 to 29 codons, characterized by interruption of perfect GAA repeats by GAG triplets. The E2 domain consisted of uninterrupted perfect GAA repeats with length variation from 5 to 11 codons (Supplemental Table S4).

### Test for neutrality

Among the non-repeat blocks of *PfGARP*, the rate of nonsynosmous substitutions per nonsynonymous site (*d*_N_ ± S.E. = 0.0086 ± 0.0038) significantly exceeded that of synonymous substitutions per synonymous site (*d*_S_ ± S.E. = 0.0000 ± 0.0000) in block 3 (*p* = 0.024) whereas no significant difference between these parameters occurred in other blocks. Meanwhile, codon based detection of deviation from selective neutrality using Fast Unconstrained Bayesian Approximation (FUBAR) has identified positive selection at codons 193 (D>Y) and 214 (Y>D) in non-repeat blocks 3 (Supplemental Table S5). Likewise, purifying selection was detected at codons 75(E) and 96(I) in block 1b and codon 678 (I) in block 13 based on the FC27 sequence.

### Phylogenetic analysis

**Figure 2:**
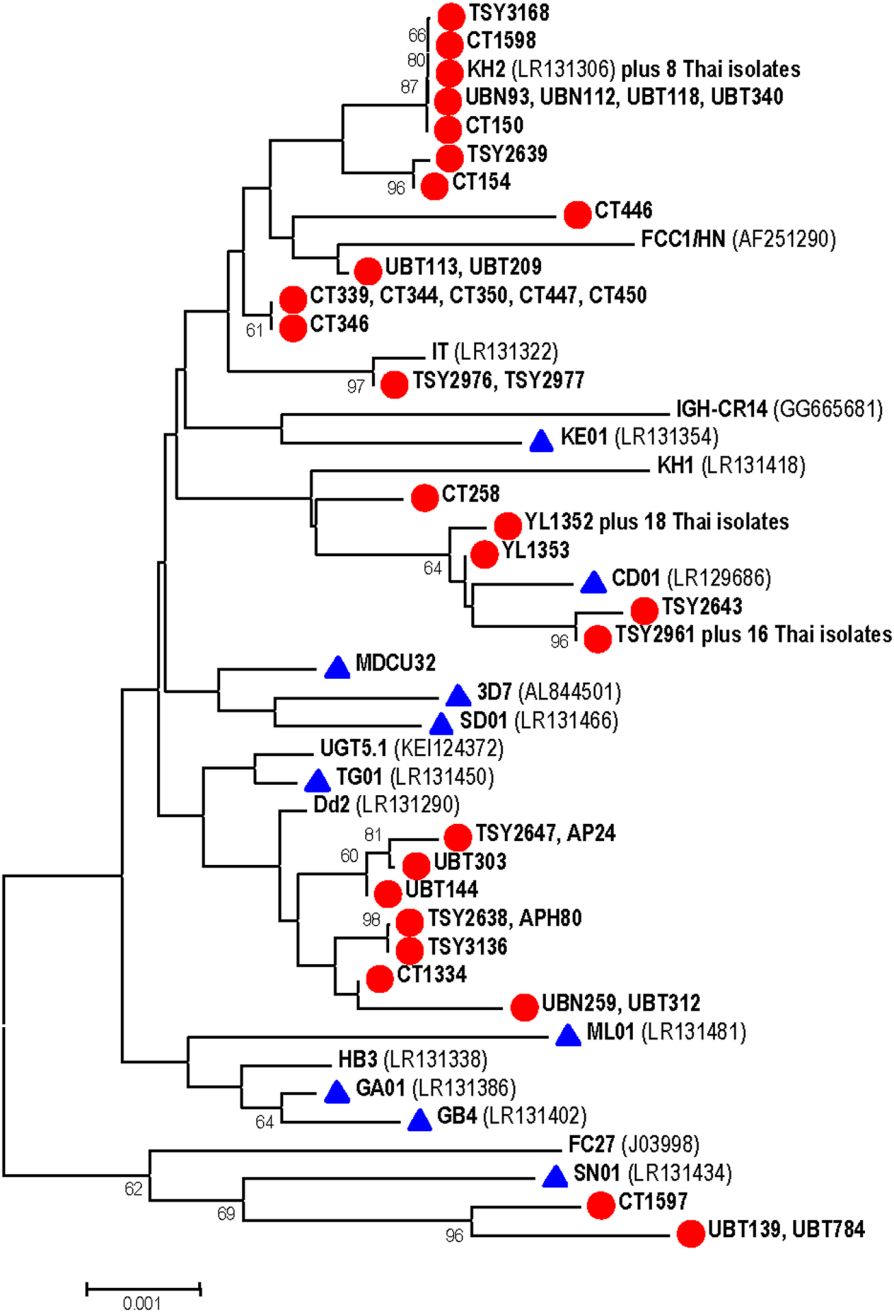
Neighbor-joining tree inferred from the complete *PfGARP* gene sequences from Thai and worldwide isolates. Thai isolates with initials TSY and AP are from Tak, UB from Ubon Ratchathani, YL from Yala and CT from Chanthaburi Provinces. The numbers following these initials are used to label individual isolates. Bootstrap values greater than 60% are shown along the branches. Thai and African isolates are marked with circles and triangles, respectively. Scale denotes nucleotide substitutions per site.

Both neighbor-joining and maximum likelihood trees inferred from the complete coding sequences of *PfGARP* did not show distinct phylogenetic clades due to the lack of high bootstrap values supporting the main branches. Like African isolates, most Thai isolates did not show any clusters or distribution based on location of origin in the phylogenetic tree. This is expected considering the described highly variable repeat domains in *PfGARP*. Out of 13 blocks, variation in repeat domains I-III, V and VI could contribute to the topology of phylogenetic tree (Fig. 2).

### Genetic differentiation

Population genetic structure inferred from allelic and genotypic frequencies of *PfGARP* was analyzed in *P. falciparum* populations from different endemic areas in Thailand by using Wright’s *F*-statistics. Almost all pairwise *F*_ST_ values among parasite populations from different endemic provinces significantly exceeded zero. However, the interpopulation variance between parasite populations from Tak and Chanthaburi Provinces was not statistically meaningful (*p* = 0.099) (Table 6).

**Table 6:** Genetic differentiation of *P. falciparum* populations inferred from *PfGARP.*

	Tak	Ubon Ratchathani	Chanthaburi	Yala
Tak		0.0090	0.0991	< 10^–5^
Ubon Ratchathani	0.1153		< 10^–5^	< 10^–5^
Chanthaburi	0.0359	0.0907		< 10^–5^
Yala	0.5526	0.5158	0.5158	

*F*_*ST*_ indices and their respective *p* values are in lower and upper diagonals, respectively.

### Parasitemia and PfGARP alleles

To determine whether variation in the number of amino acid residues in repeat regions of *PfGARP* was associated with parasitemia of the patients, analysis was performed using 76 isolates (Tak, n = 19; Ubon Ratchathani, n = 19, Chanthaburi, n = 18 and Yala, n = 20) whose parasite density could be determined. Of these, parasitemia ranged from 200 to 864,000 parasites per μL (median, 11,100 parasites/μL; geometric mean, 10,588 parasites/μL). Results revealed a tendency towards higher parasite density in patients infected with *P. falciparum* bearing more amino acid residues in repeat domains RIII and RVI including its flanking domains E1 and E2 (Kruskall–Wallis *H* test, *p* = 0.011 and 0.0281, respectively) (Table 7). No such tendency was observed for repeat domain V (*p* = 0.098) whereas limited number of isolates in categorical data or no variation in the remaining repeat domains precluded the analysis.

**Table 7:** Length polymorphism in repeat domains of *PfGARP* and parasite density.

Repeat domain	No. codons	No. isolates	Parasite density (parasites/μL)		Kruskall–Wallis *H*	*p* value
			Range	Geometric mean		
RIII	30	20	1081–17,225	4348	11.080	0.011
	35	21	1143–354,857	14,689		
	40	7	199–200,000	9387		
	45	28	1476–864,000	16,119		
RV*	21	42	1081–200,000	7164	4.641	0.098
	23	5	199–32,743	5082		
	28	28	1476–864,000	16,987		
E1-RVI-E2#	85–87	9	1553–354,857	14,004	9.093	0.0281
	88	39	1081–119,771	7047		
	89	21	199–459,000	13,018		
	91–96	7	17,849–864,000	38,418		

*One isolate containing 35 codons was omitted from analysis.

^#^Repeats with number of isolates < 5 were combined due to extensive length polymorphism in repeat domain VI. Domains RI, RII and RIV were excluded due to insufficient categorical data.

### Predicted linear B cell epitopes

Linear B cell epitopes in PfGARP were predicted based on similarity of known epitope sequences implemented in BepiBlast web server^11^ and protein language models implemented in BepiPred-3.0^12^. In total, nine B cell epitopes were predicted by the BepiBlast method, most of which spanned repeat domains. Three of these predicted epitopes, i.e. NDKENISE, KQKKIEKE and KKQEEKEK, were perfectly conserved across isolates whose sequences were similar to known epitopes in ankyrin repeat-containing protein of *Ehrlichia chaffeensis*, spike glycoprotein of severe acute respiratory syndrome coronavirus 1 and glutathione S-transferase isozyme of *Schistosoma mansoni*, respectively (Table 8). Repeat domain III contained four predicted epitopes that possessed sequence similarity either with genome polyprotein of dengue virus or M protein of *Steptococcus pyogenes*. Furthermore, two predicted epitopes were identified in repeat domain VI (Table 8). Meanwhile, prediction based on BepiPred-3.0 has identified linear B cell epitopes mostly in conserved blocks 3 and 8. All repeat-containing domains received epitope scores below the cut-off threshold by this method (Supplemental Fig. S2). The epitope score for monoclonal antibody mAb7899 capable of killing *P. falciparum *in vitro^4^ was remarkably above the epitope threshold (the N-terminal part of block 8) albeit the sequence did not share similarity to known epitopes based on the BepiBlast method (Table 8 and Supplemental Fig. S2).

**Table 8:** Predicted linear B cell epitopes spanning 8 amino acids in PfGARP and their distribution among variant alleles.

No.	Epitope	Block*	Known epitope (IEDB ID)#	Similarity of known epitope	Prevalence among Thai isolates, n = 80 (%)	Prevalence among non-Thai isolates, n = 19 (%)
1	NDKENISE	3	EPDLEEIVSILKNDKEGISE (119567)	Ankyrin repeat-containing protein of *Ehrlichia chaffeensis*	100	100
2	KQKKIEKE	4 (RII)	ESKQKKIENEIA (1429913)	Spike glycoprotein chain A of severe acute respiratory syndrome coronavirus 1	100	100
3	KKQEEKEK	4 (RII)	KPQEEKEKITKEILNGK (32844)	Glutathione S-transferase class-mu 28 Kda isozyme of *Schistosoma mansoni*	100	100
4	HKEGEHKK	6 (RIII)	VTNHMEGEHKKLAEA (1642389) and NEEMVTNHMEGEHKK (1640001)	Genome polyprotein of dengue virus	12.5	5.26
5	EGEHKEGE	6 (RIII)	LEGEWKEGEEVQVLA (1639118) and GGWKLEGEWKEGEEV (1637720)	Genome polyprotein of dengue virus	15	63.16
6	EGEDKEGE	6 (RIII)	LEGEWKEGEEVQVLA (1639118) and GGWKLEGEWKEGEEV (1637720)	Genome polyprotein of dengue virus	21.25	10.53
7	EEEHKKEE	6 (RIII)	LFEKLDKVEEEHKKVE (1465890)	M Protein of *Streptococcus pyogenes* serotype 2.1	15	36.84
8	DEEDEDDA	11 (RVI)	MYCSFYPPDEEEEDDA (1680661)	Orf1Ab polyprotein (Pp1Ab) of severe acute respiratory syndrome-related coronavirus Tor2	–	5.26
9	AEEDEDDD	11 (RVI)	AEEEEDDDMGFGLFD (876)	Ribosomal protein P-Jl5 of *Trypanosoma cruzi*	2.5	–

*After Fig. 1.

^#^Immune epitope database and analysis resource identity document.

### Predicted helper T cell epitopes

Searching for potential helper T cell epitopes recognized by HLA class II molecules with allele frequency > 0.1 among Thai population^13^ has identified four peptides in blocks 1 and 3 of PfGARP that received peptide rank < 10 and IC_50_ < 1000 nM^14^. Three of these peptides were perfectly conserved across Thai and worldwide isolates. It is noteworthy that the four peptide variants in non-repeat block 3: (i) LLLSSPYQY, (ii) LLLSSLYQY, (iii) LLLSSPYQC and (iv) LLLSSPDQY, seemed to alter the peptide rank and IC_50_ for predicted HLA class II binding peptides, particularly amino acid substitutions in variants iii and iv (Table 9).

**Table 9:** Predicted HLA class II-binding peptides in PfGARP and distribution of variant alleles.

Block*	Peptides and variants	Prevalence (%)^#^	Common HLA in Thai population^§^	IC_50_ (nM)	Peptide rank
1	FDSITGRLL	100	DRB1*15:02	203.5	4.5
1	FLSYNICIL	100	DRB1*15:02	359.2	9.8
3	LLLSSPYQY	77.5	DRB1*12:02	394.6	7.3
	LLLSS**L**YQY	0	DRB1*12:02	302.3	5
	LLLSSPYQ**C**	22.5	DRB1*12:02	1243.9	27
	LLLSSP**D**QY	0	DRB1*12:02	1841.9	38
3	AQGGLLLSS	100	DQA1*01:01/DQB1*03:01	973.8	3.3
			DQA1*01:02/DQB1*03:01	554.3	5.4
			DQA1*01:02/DQB1*03:03	994.4	8.5

*After Fig. 1.

^#^Thai isolates.

^§^Allele frequency > 10%^13^. Other common HLA class II alleles in Thai population including DQB1*05:01 and DQB1*05:02 had IC_50_ > 1000 nM and peptide rank > 10.

## Discussion

PfGARP has been recently recognized as a potential anti-disease vaccine against falciparum malaria^3,4,8^. However, the gene encoding this protein seems to be dispensable because *PfGARP*-knockout parasites could propagate normally in vitro^15^. Our analysis did not support natural deletion of this locus because *PfGARP* could be amplified by PCR from all isolates examined, corroborating with previous whole genome sequence analysis^16^. Despite being perceived as high sequence conservation based on whole genome sequence analysis, our study has shown differential variation in repetitive sequences in this locus based on direct sequencing of *P. falciparum* clinical isolates from diverse malaria endemic areas of Thailand. With more sequences analyzed, two additional domains containing repetitive sequences (domains RII and RV) have been identified. Therefore, *PfGARP* was constituted of eight repeat blocks, two of which belonged to previously recognized homopolymeric glutamic acid-encoding domains, and five highly conserved non-repeat blocks^6^ (Fig. 1).

The number of haplotypes and the extent of nucleotide diversity of *PfGARP* were almost comparable across endemic provinces in Thailand except those from Yala Province in which only two haplotypes were identified and the nucleotide diversity was two orders of magnitude lower than those observed in other endemic provinces of the country (Table 1). Consistently, our previous analyses of genetic diversity of the genes encoding circumsporozoite protein and merozoite surface protein 2 of *P. falciparum* have shown a significant lower number of haplotypes and level of nucleotide diversity of these loci among southern parasite isolates including Yala and Narathiwat Provinces than those from Tak Province, a northwestern malaria endemic area. Likewise, the number of haplotypes and nucleotide diversity of the genes encoding apical membrane antigen-1, merozoite surface proteins 1, 4 and 5 of the sympatric *P. vivax* population from Yala was significantly lower than that of Tak. Simultaneous reduction in genetic diversity of *P. falciparum* and *P. vivax* populations from Yala Province seemed to be due to population bottlenecks in both *Plasmodium* species as a consequence of control measures during the past decades and limited trans-border migration in Yala and Narathiwat Provinces^17^. However, the *F*_ST_ value inferred from sequence variation in *PfGARP* between Tak and Chanthaburi populations was not significantly different from zero, implying no genetic differentiation between these populations. Although the reason behind this finding remains elusive, a considerable number of indigenous malaria patients in Tak and Chanthaburi (including Trat) Provinces occurred among gem miners who routinely traveled between these malaria endemic areas for their occupations while insufficient treatment were common, leading to malarial gene flow between these endemic areas^18^. It has been suggested that drug resistant *P. falciparum* strains were introduced from Thai-Cambodian border to Thai-Myanmar border corresponding to the gem trade between these areas. Although the gem trade was most active only during late 1980s and early 1990s, it could be that genetic diversity within each population could have been fixed after local introduction to each endemic area^18^.

Due to meager nucleotide substitutions in non-repeat blocks of *PfGARP*, phylogenetic tree inferred from this locus mainly represented sequence variation in repeat domains. It is noteworthy that most Thai isolates were clustered in the same or related branches while a few Thai isolates (CT1597, UBT139 and UBT784) were placed outside of most Thai lineages (Fig. 2). Meanwhile, most African isolates tend to be scattered throughout the phylogenetic tree. It has been proposed that the expansion or reduction in repeat units could stem from slipped-strand mispairing mechanism or gene conversion which has been suggested to occur in repeat sequences of several malarial genes encoding vaccine candidate antigens^19–24^. The topology of phylogenetic tree may suggest that the repeat sequences in *PfGARP* seemed to have undergone independent concerted evolution, become divergent and potentially been fixed for characteristic repeat alleles between populations with geographic isolation (i.e. Southeast Asia and Africa) while diversification of repeat sequences could incidentally generate some related alleles across geographic areas^25^. Within populations, repeat sequence similarities may evolve in concert, probably following the process of random genetic drift and molecular drive which includes DNA repair and replication mechanisms in conjunction with population genetic processes^26–28^. However, extensive expansions or contractions in repeat domains of *PfGARP* could have been constrained by intrinsic stability of the repeat structure^29,30^ and/or their functional importance^7,31,32^. For example, repetitive sequence containing identical amino acids can adopt characteristic conformations that affect protein–protein interaction^33^. Interestingly, the length of homopolymeric glutamic acid repeats in domain E1 containing stretches of GAA interrupted by GAG was approximately three times longer than the perfect GAA repeats in domain E2. It seemed likely that long perfect triplet repeats encoding the same amino acids could be affected by structural instability at the DNA level unless they were interrupted by another triplet encoding the same amino acid as previously described^29,30,34^.

It is noteworthy that repeat domains RI-RIV were rich in lysine and other positively charged residues^6^. Besides the PEXEL/HT motif in non-repeat block 1 that elicited the translocation of protein into the host cell membrane, it has been shown that the low complexity sequences encoding lysine-rich tandem repeats in RI-RIV of PfGARP have involved in protein targeting to *P. falciparum*-infected erythrocyte periphery. Furthermore, the number of lysine-rich repeat units seemed to be associated with protein targeting efficiency^7^. Importantly, a minimum of 10 lysine-repeat units in domain RI seemed to be indispensable for protein targeting to the erythrocyte periphery^7^. Our sequence analysis has identified 13 alleles in domain RI with the number of lysine-repeat units ranging from 12 to 19 units; all exceeded the minimum number required for host cell peripheral targeting function (Table 2). The perfect sequence conservation of domain RIV in PfGARP has implied functional or structural importance of the region. Based on limited number of samples in this study, the length of repeat domains III and VI had a tendency to be associated with parasite density although more samples would be required to draw a firm conclusion. However, if this would be the case, the expansion of repeat units in PfGARP could probably enhance parasite survival in malaria patients. Repeat-number polymorphism in protein-coding genes has been suggested to be influenced by selection pressure^35^. Likewise, the expansion of lysine-repeat units in PfGARP could confer selective advantage for *P. falciparum*^7^. Like erythrocyte membrane protein 1 of *P. falciparum* (PfEMP-1) and other related proteins on the surface of infected erythrocytes, PfGARP has been suggested to be associated with cytoadherence property of mature asexual blood stage parasites in order to avoid host immune destruction, especially splenic removal of abnormal and infected erythrocytes^5^.

Although the gene encoding PfGARP was cloned by screening of lambda phage expression library of *P. falciparum* with sera from Papua New Guinean adults over three decades ago, it was not until recently that the significance of this molecule has been unveiled as an important target for host antibody responses capable of protecting African children with falciparum malaria from high parasitemia and severe symptoms^4^. It has been shown that anti-PfGARP antibodies conferred parasite killing through the induction of programmed cell death as evidenced by the activation of caspase-like proteases and the fragmentation of parasite DNA of late trophozoites and schizonts. Importantly, the epitope for mAB7899 antibody conferring parasite killing in vitro has been mapped to a perfectly conserved block of the protein in which high scores for linear B cell epitopes were predicted in this region by the BepiPred 3 algorithm (Supplemental Fig. S2). Although additional B cell epitopes await further investigations, prediction of linear B cell epitopes by sequence similarity with known epitopes implemented in the BepiBlast web server have identified nine potential linear B cell epitopes spanning eight amino acids, eight of these predicted epitopes were found in repeat domains and most of which exhibited sequence variation among isolates (epitopes nos. 4–9 in Table 8). Intriguingly, sequence variation in repeat domains RIII and RVI could probably be influenced by host immune pressure.

It has been shown that cognate T cell epitopes in malarial vaccine candidate antigens play a crucial role to confer clinical protection^36,37^. Searching for common Thai HLA class II binding peptides in PfGARP has predicted four epitopes in non-repeat regions (blocks 1 and 3) of the protein, three of which were invariant across isolates. Interestingly, amino substitutions at residues 214 (Y>D) and 216 (Y>C) could abolish predicted helper T cell epitope scores recognized by a common Thai HLA class II allele DRB1*12:02 (Table 9). At the nucleotide level, the substituted epitopes exhibited *d*_N_ significantly exceeding *d*_S_, suggesting that positive selection has influenced sequence variation in block 3. Although four helper T cell epitopes have been predicted in PfGARP, potential recognition of these epitopes seemed to be limited to one or a few common HLA class II alleles/haplotypes in Thai population. Meanwhile, a recent immunoinformatic and structural approach have suggested that a vaccine construct derived from PfGARP was predicted to induce both humoral and cellular immune responses^38^. Whether genetic restriction to host immune responses could compromise PfGARP vaccine efficacy awaits further studies.

In conclusion, sequence diversity in PfGARP seems to be limited to some repeat-encoding domains whereas non-repeat regions were highly conserved albeit microheterogeneity of sequence was observed particularly in regions potentially recognized by HLA class II molecules. With limited number of isolates analyzed, it seemed that expansion or reduction of lysine-rich and glutamic acid-rich repeat regions seemed to influence parasite density of malaria patients. With high sequence conservation in non-repeat and predicted immunogenic epitope regions, it is plausible that PfGARP-derived vaccine may largely elicit strain-transcending immunity.

## Materials and methods

### Parasite isolates

Blood samples were obtained from symptomatic malaria patients who were diagnosed with *P. falciparum* infections by microscopic examinations of Giemsa-stained thin and thick blood films, using a 100 × objective. The patients attended malaria clinics or district hospitals during 2009 and 2014 in Tak, Chanthaburi, Ubon Ratchathani and Yala Provinces located in northwestern, eastern, northeastern and southern parts of Thailand, respectively (Supplemental Fig. S3). Demographic data of the patients are shown in Supplemental Table S6. All blood samples were preserved in EDTA and stored at − 40 °C until use. An isolate from a Guinean patient (isolate MDCU32) was used to validate the protocol to genotype a sample from high-transmission setting.

### DNA extraction

Two hundred microliters of EDTA-preserved blood sample from each patient were deployed for DNA extraction using Qiagen DNA mini kit (Qiagen, Hilden, Germany) following the manufacturer’s instruction. DNA samples were stored at − 40 °C until use.

### PCR detection and genotyping of *P. falciparum*

All isolates diagnosed with *P. falciparum* monoinfections by microscopy were reaffirmed by species-specific nested PCR^39^. Genotypes of *P. falciparum* were determined by size polymorphism in block 2 of the merozoite surface proteins-1 (PfMSP1) and the central repeat region of the merozoite surface proteins-2 (PfMSP2) as described previously^40^. Isolates yielding single bands of both PfMSP1 and PfMSP2 on agarose gel electrophoresis were included for further analysis. In total 80 isolates were used in this study, consisting of 20 isolates from each endemic province (Supplemental Fig. S3).

### Parasite density

Estimation of parasite density was done from at least 200 white blood cells in Giemsa-stained thick blood films, using a 100 × objective. The procedure was performed by a well-trained microscopist with > 20 years of experience in detection and identification of malaria parasite species. Parasite density was determined twice using duplicated blood films from each patient.

### PCR amplification and sequencing of the PfGARP gene

The complete coding sequence of *PfGARP* was amplified by PCR using primers PfGARP-F0 (5′-ATAAATAAAGATTAGTATATTTAAAACG-3′) and PfGARP-R0 (5′-AAATAGCTTTGATTTAACACATTAC-3′). DNA amplification was carried out in a total volume of 20 µL containing 2 µL of DNA template, 2.5 mM each deoxynucleoside triphosphate, 3 μL of 10 × PCR buffer, 0.3 μM of each primer and 1.25 unit of ExTaq DNA polymerase (Takara, Seta, Japan). The PCR thermal profile included a preamplification denaturation at 94 °C for 1 min, 35 cycles of 94 °C for 40 s, 50 °C for 30 s and 72 °C for 3 min, and a final extension at 72 °C for 10 min. Amplicons were analyzed by 1% agarose gel electrophoresis, stained with ethidium bromide and visualized under UV transilluminator. Sequences were determined directly and from both directions using the PCR-purified products as templates and sequencing primers (Supplemental Table S7). Singletons and unique insertion-deletion of sequences were verified by re-sequencing of the PCR products from independent amplification reactions using the same genomic DNA as templates.

### Data analysis

Sequence analysis included 80 nucleotide sequences of *PfGARP* from Thai isolates, one clinical isolate from Guinea (isolate MDCU32) and 18 publicly available complete gene sequences whose isolate names, country of origins and their GenBank accession numbers are as follows: 3D7 (Netherlands from West Africa, AL844501), CD01 (Congo, LR129686), Dd2 (Indochina, LR131290), FC27 (Papua New Guinea, J03998), FCC1/HN (Hainan in China, AF251290), GA01 (Gambia, LR131386), GB4 (Ghana, LR131402), KH1 (Cambodia, LR131418), KH2 (Cambodia, LR131306), HB3 (Honduras, LR131338), IGH-CR14 (India, GG6656811), IT (Brazil, LR131322), KE01 (Kenya, LR131354), ML01 (Mali, LR131481), SD01 (Sudan, LR131466), SN01 (Senegal, LR131434), TG01 (Togo, LR131450), and UGT5.1 (Vietnam, KE124372). Of these, the 3D7, FC27and FCC1/HN sequences were determined by Sanger dideoxy-chain termination method whereas the remaining isolates were assembled sequences from next-generation sequencing platforms (Supplemental Table S1). Sequence alignment was performed by using the CLUSTAL_X program, taken into account appropriate codon match in the coding region by manual adjustment to maintain the reading frame. The sequence from the FC27 strain was used as a reference^6^. Searching for nucleotide repeats was performed by using the Tandem Repeats Finder version 4.0 program with the default option. Nucleotide diversity (π), the rate of synonymous substitutions per synonymous site (*d*_S_) and the rate of nonsynonymous substitutions per nonsynonymous site (*d*_N_) were determined from the average values of sequence differences in all pairwise comparison of each taxon and the standard error was computed from 1000 bootstrap pseudoreplicates implemented in the MEGA 6.0 program^41^. Haplotype diversity and its sampling variance were computed by taking into account the presence of gaps in the aligned sequences using the DnaSP version 5.10 program^42^. Natural selection on codon substitution was determined by using fast unconstrained Bayesian approximation (FUBAR) method in the Datamonkey Web-Server^43,44^. Neighbor-joining phylogenetic tree based on nucleotide sequences was constructed by using maximum composite likelihood parameter whereas maximum likelihood tree was built using Tamura-Nei model with the rate variation model allowed for some sites to be evolutionarily invariable. The Arlequin 3.5.2.2 software was deployed to determine genetic differentiation between populations, the fixation index (*F*_ST_), using analysis of molecular variance approach (AMOVA) akin to the Weir and Cockerham’s method but taken into account the number of mutations between haplotypes^45^. One hundred permutations were deployed to determine the significance levels of the fixation indices. Prediction of linear B cell epitopes in *PfGARP* was performed by using a sequence similarity to known experimentally verified epitopes from the Immune Epitope DataBase (IEDB) implemented in the BepiBlast Web Server^11^. Furthermore, linear B cell epitopes were also predicted based on protein language models implemented in BepiPred-3.0^12^. Potential HLA-class II-binding peptides were analyzed by using the IEDB recommended 2.22 algorithm with a default 12–18 amino acid residues option. The predicted HLA-class II-binding peptides were predicted based on the percentile rank < 10 and the IC_50_ threshold for HLA binding affinity ≤ 1000 nM^14^. The analysis mainly concerned the common HLA class II haplotypes among Thai populations with allele frequency > 0.1^13^.

### Ethical approval

This study was reviewed and approved by the Institutional Review Board in Human Research of Faculty of Medicine, Chulalongkorn University, Thailand (IRB No. 193/64; COA No. 468/2021). Prior to blood sample collection, written informed consent was obtained from all participants or from their parents or guardians. All procedures were performed in accordance to the relevant guidelines and regulations.

### Accession numbers

Eighty-one complete sequences of the *PfGARP* gene of *Plasmodium falciparum* have been deposited in NCBI GenBank under accession numbers OQ197883-OQ197963.

## Supplementary Information

Supplementary Information.

## Data Availability

The datasets generated during and/or analyses during the current study are available from the corresponding authors upon request.
