# Supplementary material for: Analysis of sequence diversity in Plasmodium falciparum glutamic acid-rich protein (PfGARP), an asexual blood stage vaccine candidate
Source: Sci Rep. 2023 Mar 9;13:3951. doi: 10.1038/s41598-023-30975-4 (PMC9996596; doi:10.1038/s41598-023-30975-4)

Analysis of sequence diversity in *Plasmodium falciparum* glutamic acid-rich protein (PfGARP), an asexual blood stage vaccine candidate

Rattanaporn Rojrung[1,2], Napaporn Kuamsab[1,3], Chaturong Putaporntip[1,*], Somchai Jongwutiwes[1,*]

[1]Molecular Biology of Malaria and Opportunistic Parasites Research Unit, Department of Parasitology, Faculty of Medicine, Chulalongkorn University, Bangkok, Thailand;

[2]Medical Sciences Program, Faculty of Medicine, Chulalongkorn University, Bangkok, Thailand;

[3]Community Public Health Program, Faculty of Health Science and Technology, Southern College of Technology, Nakorn Si Thammarat

SUPPLEMENTAL INFORMATION

Contents

Table S1 Complete PfGARP genes from public database……………………………………...…2

Table S2 Nucleotide substitutions in non-repeat blocks of *PfGARP* ….……….…………….....3

Table S3 Diversity and distribution of repeat alleles in block 4 (repeat domain II) of PfGARP…...………………………………………………………………….....4

Table S4 Sequence variation in polyglutamic acid repeats in E1 and E2 domains of *PfGARP*……………….…………………………………………….……………......5

Table S5 Codons under positive and negative selection in *PfGARP*…..…...…………….........6

Table S6 Demographic data of malaria patients……..……………………………………...7

Table S7 Sequencing primers for *PfGARP* …..……...………………………………...…8

Figure S1 Distribution of *PfGARP* haplotypes inferred from coding regions among 80 Thai isolates…….……………………………………..………..…..……9

Figure S2 Linear B cell epitope scores across PfGARP predicted by BepiPred version 3.0..…10

Figure S3 Map of Thailand showing sample collection sites………..………..…...……..……11

Supplemental Table S1 Complete PfGARP genes from public database

Isolate /strain	GenBank accession no.	Complete gene (bp)	Coding region (bp)	Intron (bp)	Geographic origin	Sequencing method
3D7	AL844501	2233	2019	214	Netherlands from West Africa	Sanger sequencing from contigs
CD01	LR129686	2221	2007	214	Congo	Next-generation sequencing platform
Dd2	LR131290	2244	2028	216	Indochina	Next-generation sequencing platform
FC27	J03998	2248	2034	214	Papua New Guinea	Sanger sequencing from plasmid clones
FCC1/HN	AF251290	2260	2046	214	Hainan, China	Sanger sequencing from plasmid clones
GA01	LR131386	2266	2052	214	Gambia	Next-generation sequencing platform
GB4	LR131402	2250	2034	216	Ghana	Next-generation sequencing platform
HB3	LR131338	2224	2010	214	Honduras	Next-generation sequencing platform
IGH-CR14	GG665681	2245	2031	214	India	Next-generation sequencing platform
IT	LR131322	2248	2034	214	Brazil	Next-generation sequencing platform
KE01	LR131354	2257	2043	214	Kenya	Next-generation sequencing platform
KH1	LR131418	2251	2037	214	Cambodia	Next-generation sequencing platform
KH2	LR131306	2266	2052	214	Cambodia	Next-generation sequencing platform
ML01	LR131481	2244	2028	216	Mali	Next-generation sequencing platform
SD01	LR131466	2248	2034	214	Sudan	Next-generation sequencing platform
SN01	LR131434	2254	2040	214	Senegal	Next-generation sequencing platform
TG01	LR131450	2248	2034	214	Togo	Next-generation sequencing platform
UGT5.1	KE124372	2209	1995	214	Vietnam	Next-generation sequencing platform

Supplemental Table S2 Nucleotide substitutions in nonrepeat blocks of *PfGARP*

Codon (Block)	Nucleotide substitution (amino acid)	Thai isolates, n					Non-Thai isolates/strains #	Total
		Tak	Ubon Ratchathani	Chanthaburi	Yala	Total		
75 (1b)	GAA (E)	20	20	20	20	80	3D7, CD01, Dd2, FC27, GA01, GB4, KH1, KH2, HB3, IGH-CR14, IT, KE01, ML01, SD01, SN01, TG01, UGT5.1 and MDCU32.	18
	GAG (E)	0	0	0	0	0	FCC1/HN	1
96 (1b)	ATA (I)	0	0	0	0	0	FC27	1
	ATT (I)	20	20	20	20	80	3D7, CD01, Dd2, FCC1/HN, GA01, GB4, KH1, KH2, HB3, IGH-CR14, IT, KE01, ML01, SD01, SN01, TG01, UGT5.1 and MDCU32.	18
165 (3)	AAA (K)	20	20	20	1	61	CD01, Dd2, FC27, FCC1/HN, KH1, KH2, IGH-CR14, IT, KE01 and UGT5.1.	10
	GAA (E)	0	0	0	19	19	3D7, GA01, GB4, HB3, ML01, SD01, SN01, TG01 and MDCU32.	9
193 (3)	GAT (D)	0	0	0	0	0	CD01, FC27, KE01, SD01 and UGT5.1.	5
	TAT (Y)	20	20	20	20	80	3D7, Dd2, FCC1/HN, GA01, GB4, KH1, KH2, HB3, IT, IGH-CR14, ML01, SN01, TG01 and MDCU32.	14
213 (3)	CCA (P)	20	20	20	20	80	3D7, CD01, Dd2, FC27, FCC1/HN, GA01, GB4, KH1, KH2, HB3, IGH-CR14, KE01, ML01, SD01, SN01, TG01, UGT5.1 and MDCU32.	18
	CTA (L)	0	0	0	0	0	IT	1
214 (3)	TAT (Y)	20	20	20	20	80	3D7, CD01, Dd2, FC27, FCC1/HN, GB4, KH1, KH2, HB3, IGH-CR14, IT, KE01, SD01, SN01, TG01 and UGT5.1.	16
	GAT (D)	0	0	0	0	0	GA01, ML01 and MDCU32.	3
216 (3)	TAT (Y)	10	18	14	20	62	3D7, CD01, Dd2, FC27, FCC1/HN, GA01, GB4, KH1, KH2, HB3, IGH-CR14, IT, KE01, ML01, SD01, SN01, TG01, UGT5.1 and MDCU32.	19
	TGT (C)	10	2	6	0	18	-	0
678 (13)	ATC (I)	10	2	7	20	39	CD01, FC27 and FCC1/HN.	3
	ATT (I)	10	18	13	0	41	3D7, Dd2, GA01, GB4, KH1, KH2, HB3, IGH-CR14, IT, KE01, ML01, SD01, SN01, TG01, UGT5.1 and MDCU32.	16

GenBank accession numbers are listed in Supplemental Table S1.
MDCU32 is from a Guinean patient.
Dash denotes none among non-Thai isolates/strains.

Supplemental Table S3 Diversity and distribution of repeat alleles in block 4 (repeat domain II) of PfGARP

Allele	Amino acid sequence	Thai isolates, n					Non-Thai isolates/strains*	Total
		Tak	Ubon Ratchathani	Chanthaburi	Yala	Total		
RII-73	KKERKQKEKEMK**E**QEKIEKK**K**KK**Q**EEKEKKK**QE**<br>KERKKQE<br>KKERKQKEKEMK**K**QKKIEKE**R**KK**K**EEKEKKK**KK**	0	0	0	0	0	FC27, 3D7, SD01 and CD01	4
RII-71	KKERKQKEKEMK**E**QEKIEKK**K**KK**Q**EEKEKKK**QE**<br>KERKKQE<br>KKERKQKEKEMK**K**QKKIEKE**R**KK**K**EEKEKKK--	20	20	20	20	80	Dd2, FCC1/HN, GA01, GB4, HB3, IGH-CR14, IT, KE01, KH1, KH2, MDCU32, ML01, SN01, TG01 and.UGT5.1	15
	Total	20	20	20	20	80		19

* GenBank accession numbers are listed in Supplemental Table S1.
MDCU32 is from a Guinean patient.

Supplemental Table S4 Sequence variation in polyglutamic acid repeats in E1 and E2 domains of *PfGARP*

Domain/ Allele	Sequence#	Thai isolates					Non-Thai isolates/strains*	Total
		Tak	Ubon Ratchathani	Chanthaburi	Yala	Total		
E1								
E1-29	11111112111121121111211112111	10	2	6	20	38	FC27, CD01	2
E1-27	111111112111121111211112111	4	2	0	0	6	-	0
E1-26	11111111121111211112111121	0	0	0	0	0	IGH-CR14	1
E1-24	111111121111211112111121	0	0	0	0	0	GA01, SN01	2
E1-23	11111112111111111112111	0	0	0	0	0	SD01	1
E1-21A	111111121111111112111	0	0	0	0	0	3D7	1
E1-21B	111111121111211112111	0	0	11	0	11	UGT5.1, Dd2, KH1, ML01, TG01	5
E1-21C	111111111111211112111	0	2	0	0	2	-	0
E1-19	1111111211112111121	0	0	0	0	0	KE01	1
E1-16	1111111211112111	6	14	3	0	23	FCC1/HN, KH2, GB4, HB3, IT, MDCU32	6
Total		20	20	20	20	80		19
E2								
E2-11	11111111111	0	0	0	0	0	KE01	1
E2-10	1111111111	0	0	1	0	1	FC27, Dd2	2
E2-9	111111111	10	2	7	20	39	ML01, CD01	2
E2-8	11111111	0	0	9	0	9	KH1, MDCU32	2
E2-7	1111111	6	18	3	0	27	FCC1HN, KH2, IT	3
E2-6	111111	0	0	0	0	0	3D7, IGH-CR14, SD01, GA01, SN01	5
E2-5	11111	4	0	0	0	4	UGT5.1, TG01, GB4, HB3	4
Total		20	20	20	20	80		19

1 and 2 represent GAA and GAG, respectively. Alleles are assigned based on the number of codons and their variants in alphabetical orders.
* GenBank accession numbers are listed in Supplemental Table S1.
MDCU32 is from a Guinean patient.
Dash denotes none among non-Thai isolates/strains.

Supplemental Table S5 Codons under positive and negative selection in *PfGARP*

Codon#	Block	α	β	β - α	Posterior probability [α > β]	Posterior probability [α < β]	Bayes Factor [α < β]	Selection*
75 (GAA → GAG)	1b	25.01	1.20	-23.81	0.928	0.055	0.076	Negative
96 (ATA → ATT)	1b	24.56	1.10	-23.46	0.928	0.056	0.078	Negative
193 (GAT → TAT)	3	4.32	39.96	35.64	0.018	0.947	23.379	Positive
214 (TAT → GAT)	3	4.27	31.76	27.49	0.041	0.921	15.326	Positive
678 (ATC → ATT)	13	38.23	1.09	-37.14	0.993	0.003	0.004	Negative

# Positions are corresponding to coding sequence of the FC27 strain, GenBank accession no. J03998).
* Posterior probabilities > 0.9.

Supplemental Table S6 Demographic data of malaria patients

Characteristiscs	**Province** (%)				**Total** n = 80
	**Tak** n = 20	**Ubon Ratchathani** n = 20	**Chanthaburi** n = 20	**Yala** n = 20	
**Gender**					
Male	14 (70)	20 (100)	16 (80)	12 (60)	62 (77.50)
Female	6 (30)	0 (0.00)	4 (20)	8 (40)	18 (22.50)
**Age** (year)					
< 20	12 (60)	4 (20)	8 (40)	9 (45)	33 (41.25)
20 – 40	6 (30)	14 (70)	10 (50)	10 (50)	40 (50.00)
> 40	2 (10)	2 (10)	2 (10)	1 (5)	7 (8.75)
Range	6 - 50	17 - 45	10 - 78	6 - 64	6 - 78
Mean ± S.D.	19.85 ± 10.52	26.60 ± 6.73	28.50 ± 11.38	24.10 ± 9.56	24.76 ± 12.92
**Ethnicity**					
Thai	2 (10)	19 (95)	10 (50)	20 (100)	51 (63.75)
Cambodia*	0	0	10 (50)	0	10 (12.50)
Karen*	18 (90)	0	0	0	18 (22.50)
Laos*	0	1 (5)	0	0	1 (1.25)

* Local settlements in Thailand and transmigration.

Supplemental Table S7 Sequencing primers for *PfGARP*

Primer	Sequence (5'→3')	Direction	Positions*
PFGARPF02	GATTAGTATATTTAAAACGTAATATA	Forward	(-75) - (-57)
PFGARPF01	AATGAATGTGCTATTTCTTTCGTAT	Forward	(-1) - 24
PfGARPF1	CGGGTCAACATAAACCAAAAAACGC	Forward	741 - 765
PfGARPF2	ACACGTAGTTAAAAATGTTATAGAAGA	Forward	1534 - 1560
PFGARPR1	AAATATATTAAAAAAGGGATGG	Reverse	2248 - 2269
PFGARPR2	TACATTGTGGTTGGCTTAGTGGTCTAC	Reverse	1638 - 1664
PFGARPR3	ACTAGATATGATTCCACATCCTCC	Reverse	872 - 895
PFGARPR4	ACAGAGTTTTCATTATCTTTATC	Reverse	542 - 564
PFGARPR5	CAAGTGTTTTCAACATTTCTTC	Reverse	974 - 995

*after the FC27 sequence (GenBank accession no. J03998).

Supplemental Figure S1 Distribution of *PfGARP* haplotypes inferred from coding regions among 80 Thai isolates

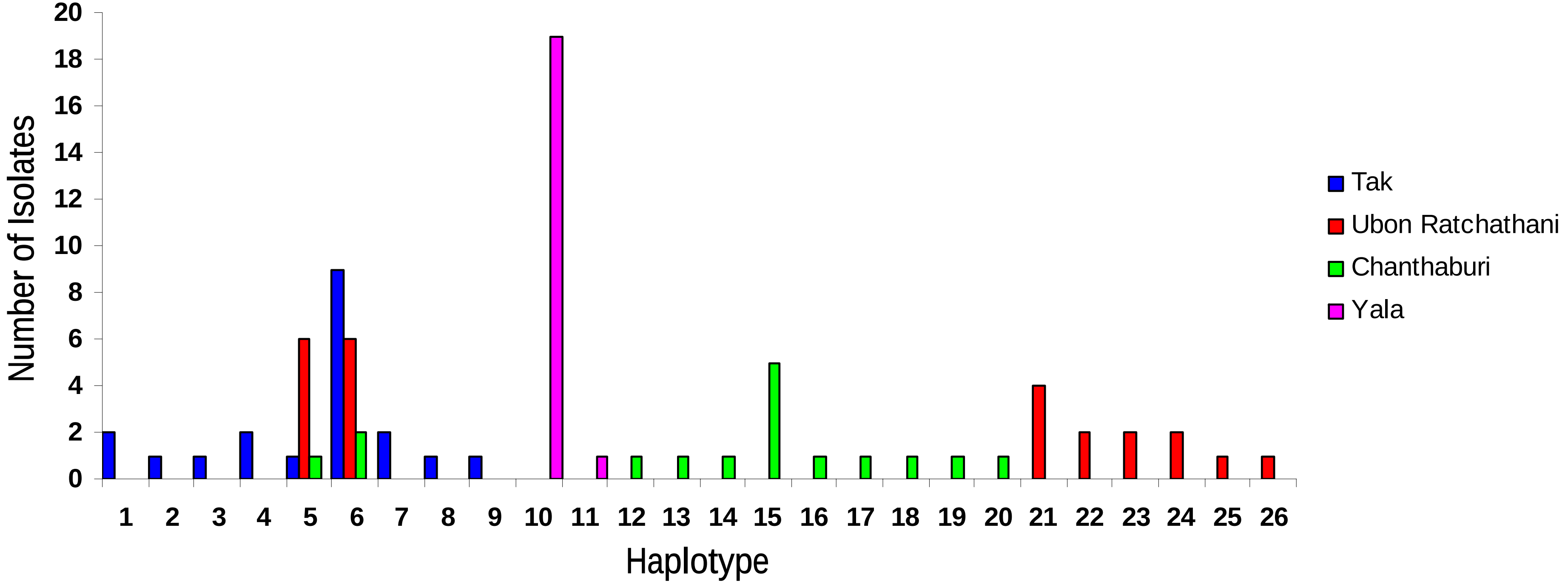

Supplemental Figure S2 Linear B cell epitope scores across PfGARP predicted by BepiPred version 3.0. Blocks are after Fig. 1. Red line represents epitope threshold. Green and blue boxes indicate ligand for erythrocyte band 3 and epitope for mAb7899, respectively.

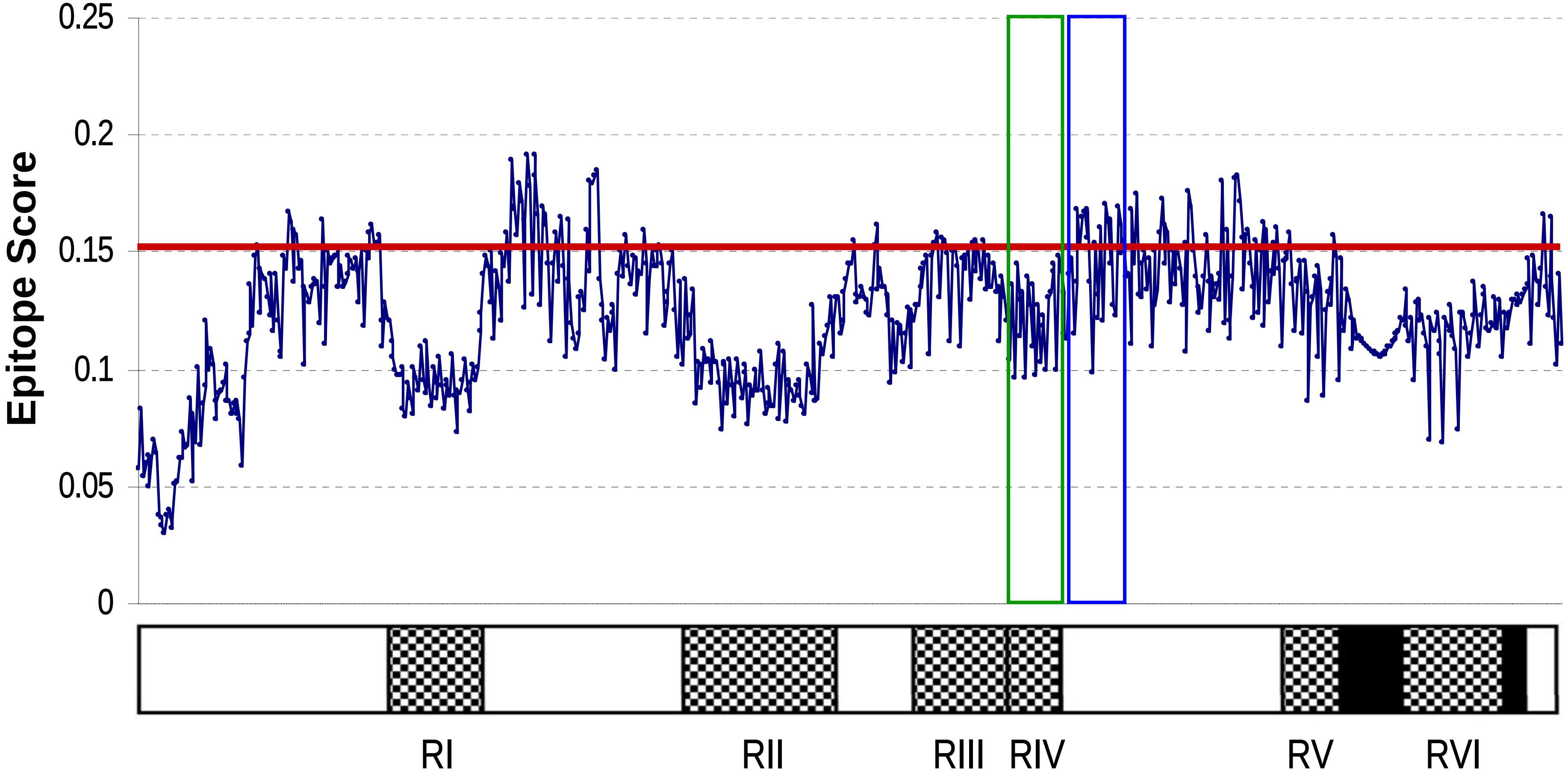

Supplemental Figure S3 Map of Thailand showing sample collection sites. The map is modified from GADM maps and data (https:// gadm.org/ index. html) under the GADM license version 6.0.

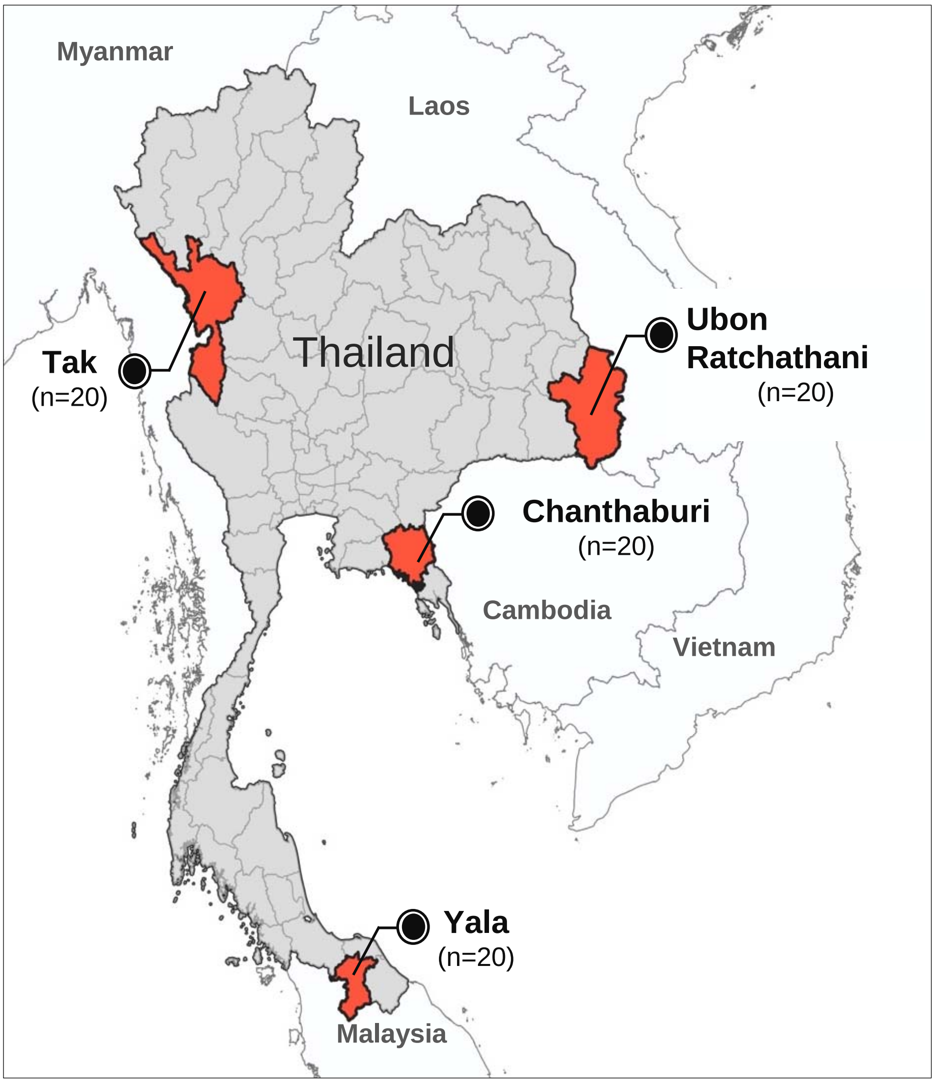

Supplement: Supplementary file 1 — Supplementary Information. [file 41598_2023_30975_MOESM1_ESM.pdf]
